# Everybody's Page

**Published:** 1892-08-06

**Authors:** 


### EVERYBODY'S PAGE.

One of the prettiest sights at the Chicago World's Fair should be the yachts. It is stated that the principal yacht clubs on the lakes and sea coast will be represented by several hundred yachts.

The *New York Medical Record* has discovered one thing about which doctors agree! All physicians in the Paris hospitals believe in the efficacy of the action of salicylate of soda in attacks of acute rheumatism. But alas, their agreement ends here; in the mode of administration their differences are considerable.

The possibilities of cod-liver oil milk in the field of medicine have been referred to by us. Another combination, more palatable, in the form of eucalyptus honey, or honey made by bees which cull from the eucalyptus flower, is advocated by some as a remedy for the treatment of pulmonary phthisis. There is no reason to doubt that it possesses useful antiseptic properties.

Doing good sometimes brings worse than a barren reward. It has becided by a French court that a person who summons a doctor to the bedside of a sick person is responsible for the payment of his fees. As the decision presumably arose out of a case where a doctor had been called in to a poor person by someone who could afford to pay the fees, the justice of the decision can hardly be disputed. Good Samaritans cannot expect to do all their good actions for nothing.

A writer in this month's *Harper's* draws attention to the enormous consumption in New York of ice taken from contaminated sources. The picture he draws is not a pleasant one for the inhabitants of that city to contemplate. The great ice supply is drawn from the neighbourhood of Troy and Albany on the Upper Hudson, where "typhoid fever is nearly always present during the ice-harvesting season. We know that the waste from these victims of disease is cast into the Hudson River. We know that the typhoid germ resists freezing and long-continued cold, and yet between seven and eight hundred thousand tons of ice are cut from the Hudson in average years within twelve miles of Albany largely for the refreshment of New Yorkers." If this is so it is of little avail that so much energy should have been expended in procuring an almost perfect water supply, the benefit of which is neutralized in such a deadly manner.

There is a well-marked distinction, according to a French savant, between drunkenness and inebriety. The former is a vice; the latter is a disease. "Drunkards are people who drink whenever they find an opportunity for drinking; inebriates drink only when the attack seizes them." This seems rather a distinction without a difference; an inebriate is usually "attacked" whenever he gets an opportunity.

The proposal to tax bachelors in France will, if carried into effect, probably produce somewhat complicated results, and it is not beyond the range of probability that the results may be the reverse of those looked for. The confirmed bachelor will perhaps consider the tax the lesser of two evils, though it sounds ungallant to say so. The imposition of the tax will also place the woman in an unenviable position; to a certain extent she will return to her position of the middle ages, in so far that as she will become an alternative, of course a pleasant one to all except the most wrong-minded of men, she will thereby become a species of chattel. Put the proposition in any light you will, it is not a graceful one, nor is it altogether complimentary to the fair sex or in keeping with the best traditions of the French.

The recent riots and attacks on doctors in Russia show very clearly how widespread superstition is in that country. Though the peasants appear to have a rooted dislike to the properly qualified practitioner, there is a popular kind of quackery in which they place unlimited faith. Two great remedies for intermittent fever are frogs and fright; it is, perhaps, superfluous to say that the latter is looked upon with greater favour as the more efficacious of the two. As soon as the fever manifests itself the patient's pockets are filled with as many live frogs as they will hold. With nervous people there is, of course, sufficient admixture of fright to make the cure doubly certain, but if this cure fails with a strong-minded sufferer his fellow-villagers attempt fright. Their kindly anxiety for their victim is shown in a very remarkable manner. A crowd of them enter his hut and raise a quarrel with him, calling him offensive names. Being strong-minded, he naturally retorts, to which his kind friends object. Force is called in to their aid, they seize the patient, tie a rope round his neck, and drag him about till he becomes unconscious from fright. It is not stated that this method kills more than it cures; but it has its advantages, as it must do one or the other.

